# Serotonergic modulation of ‘waiting impulsivity' is mediated by the impulsivity phenotype in humans

**DOI:** 10.1038/tp.2016.210

**Published:** 2016-11-08

**Authors:** S Neufang, A Akhrif, C G Herrmann, C Drepper, G A Homola, J Nowak, J Waider, A G Schmitt, K-P Lesch, M Romanos

**Affiliations:** 1 Center of Mental Health, Department of Child and Adolescent Psychiatry, Psychosomatics and Psychotherapy, University of Wuerzburg, Wuerzburg, Germany; 2 Department of Neuroradiology, University of Wuerzburg, Wuerzburg, Germany; 3 Department of Radiology, University of Wuerzburg, Wuerzburg, Germany; 4 Center of Mental Health, Division of Molecular Psychiatry, Department of Psychiatry, Psychosomatics and Psychotherapy, University of Wuerzburg, Wuerzburg, Germany; 5 Center of Mental Health, Department of Psychiatry, Psychosomatics and Psychotherapy, University of Wuerzburg, Wuerzburg, Germany

## Abstract

In rodents, the five-choice serial reaction time task (5-CSRTT) has been established as a reliable measure of waiting impulsivity being defined as the ability to regulate a response in anticipation of reinforcement. Key brain structures are the nucleus accumbens (NAcc) and prefrontal regions (for example, pre- and infralimbic cortex), which are, together with other transmitters, modulated by serotonin. In this functional magnetic resonance imaging study, we examined 103 healthy males while performing the 5-CSRTT measuring brain activation in humans by means of a paradigm that has been widely applied in rodents. Subjects were genotyped for the tryptophan hydroxylase-2 (*TPH2*; G-703T; rs4570625) variant, an enzyme specific for brain serotonin synthesis. We addressed neural activation patterns of waiting impulsivity and the interaction between the NAcc and the ventromedial prefrontal cortex (vmPFC) using dynamic causal modeling. Genetic influence was examined via interaction analyses between the *TPH2* genotype (GG homozygotes vs T allele carriers) and the degree of impulsivity as measured by the 5-CSRTT. We found that the driving input of the vmPFC was reduced in highly impulsive T allele carriers (reflecting a reduced top-down control) in combination with an enhanced response in the NAcc after correct target processing (reflecting an augmented response to monetary reward). Taken together, we found a high overlap of our findings with reports from animal studies in regard to the underlying cognitive processes, the brain regions associated with waiting impulsivity and the neural interplay between the NAcc and vmPFC. Therefore, we conclude that the 5-CSRTT is a promising tool for translational studies.

## Introduction

Waiting impulsivity (WI), compared with common impulsivity measures such as motor response inhibition,^1^ delay discounting^2^ and reflection impulsivity,^3^ is defined operationally as the tendency to premature responding, that is, to respond before target onset. WI can be assessed using the five-choice serial reaction time task (5-CSRTT),^4, 5^ which involves aspects of response inhibition, mediated by motivational aspects. The paradigm is based on the human continuous performance task^6^ and employs measures of sustained attention and action restraint while awaiting a reward. Premature responses are assumed to arise as a consequence of the individual expecting a reward-related cue in combination with aspects of response inhibition. To date, the 5-CSRTT has mainly been employed in rodents^7^ with only three human behavioral studies.^8, 9, 10^

In electrophysiological studies in rodents, WI has been associated with the prefrontal cortex (PFC) including the anterior cingulate cortex (ACC),^11^ the dorsal and ventral prelimbic cortices^12^ (human homolog: dorsal cingulate cortex, Brodmann Area 32), and the infralimbic cortex (human homolog: ventromedial PFC (vmPFC), Brodmann Area 25) interacting with mediotemporal structures such as the hippocampus and the amygdala, and the nucleus accumbens (NAcc).^6, 13^ This network is strongly modulated by neurotransmitters of dopaminergic neurons in the ventral tegmental area, serotonergic neurons in the raphe nuclei and noradrenergic neurons in the locus coeruleus.^4, 5, 6, 13^ The best examined structures, to date, are the NAcc in combination with the vmPFC, with regard to their functional interaction while performing the 5-CSRTT. For example, Donnelly *et al.* examined rats while performing the 5-CSRTT and reported that gamma frequency (50–60 Hz) in local field potential oscillations transiently increased in the vmPFC and NAcc during the waiting period and after the performance of a correct response. The first finding has been discussed to presumably reflect increasing top-down control demands over waiting time^14^ and the second finding being associated with the processing of reward.^14, 15^ Highly impulsive rats (animals with high number of premature responses) showed reduced activity during the waiting period^16^ predominantly in the vmPFC, hinting towards an impaired top-down control in highly impulsive animals.

The relation between activity in the vmPFC and premature responding has been demonstrated in a lesion study by Christakou *et al.* Disconnection of the vmPFC and the NAcc led to increased impulsive behavior.^17^ In pharmacological studies, the transient inactivation of the vmPFC by injection of the γ-aminobutyric acid receptor agonist led to the dose-specific effects on behavioral performance, whereas low doses impaired impulse control indicated by heightened premature responding, high doses of muscimol induced deficits in impulse and attentional control in 5-CSRTT performance.^18, 19, 20^ The pharmacological inactivation of the NAcc, in return, impaired general task performance in terms of impulse control deficits (accuracy) and severe general impairments in task performance (for example, slower reaction times, RT). Thus, the vmPFC may be considered as one crucial structural correlate for impulsivity and response inhibition, whereas the NAcc may have a relevant role in the prevention of premature response during anticipation of reward.^18, 19, 20^

Serotonergic modulation of WI has been examined in both humans and rodents. Several animal studies investigated the impact of serotonergic neurotransmission on WI revealing region-specific modulations. Although 5-HT depletion in the NAcc did not affect behavioral parameters,^21^ the administration of 5-HT2A and 5-HT2C antagonists within the NAcc had opposite effects with 5-HT2A blocking and 5-HT2C increasing impulsivity.^21^ The administration of 5-HT2A and 5-HT1A receptor agonist in vmPFC regions, however, significantly enhanced target detection^22^ and reduced the number of premature responses.^23^ Serotonergic modulation of WI in humans has been examined in the study by Worbe *et al.*^9^ using a tryptophan depletion (TD) approach. In contrast to region-specific serotonergic manipulation in rodents, this approach addresses an overall effect of serotonin reduction. They found that TD significantly increased the number of premature responses. However, this increase varied in function of the subject's trait impulsivity as measured by the motor impulsivity subscale of the Barratt Impulsivity Scale, suggesting an interaction between serotonergic modulation and individual impulsivity: the more impulsive TD subjects were the higher the number of premature responses they committed. In addition, tryptophan-depleted participants demonstrated a higher motivational index compared with non-depleted subjects^9^ hinting towards a serotonergic modulation not only of measures of impulsivity^24^ but also of motivation and reward processing.

To our knowledge, this is the first study that presents the neural data of humans while performing the 5-CSRTT. In this pilot study, we examined the neural underpinnings of WI as measured by the 5-CSRTT in humans using functional neuroimaging aiming to replicate the neural findings so far as presented on the network level by Dalley *et al.*^6^ as well as on the interaction between the key structures vmPFC and the NAcc by Donelly *et al.* and Feja *et al.*^18^ We examined 103 young male subjects using a magnetic resonance imaging-adapted version of the human 4-CSRTT as suggested by Voon *et al.*^8^ Based on the named findings, we focused on the interplay between the key structures NAcc and vmPFC in terms of brain activation and effective connectivity between both structures. Effective connectivity was determined using dynamic causal modeling (DCM).^25^ Based on the findings that top-down demands increase within waiting time, we expected an increase of vmPFC recruitment at the beginning of the waiting period and strongest vmPFC activation during the target condition. The NAcc was expected to be active in the anticipation of reward, starting in the ‘target' condition, and during reward receipt, as defined in the ‘reward' condition.

In a second step, we addressed the serotonergic modulation of NAcc and vmPFC connectivity in terms of analyzing a tryptophan hydroxylase-2 gene variant (*TPH2*; G-703T; rs4570625). TPH2 is brain-specific serotonin synthesizing enzyme; the variant has been shown to affect emotional and non-emotional processing of the amygdala and within cortico-striatal circuits.^26, 27^ TPH is an enzyme involved in the synthesis of serotonin. *TPH2* is the brain-specific isoenzyme of TPH and is primarily expressed in the serotonergic neurons of the brain localized in the raphe nuclei, which project to numerous brain regions including the hypothalamic nuclei,^28^ the striatum^27, 28^ and in mediotemporal structures hippocampus and amygdala,^27, 29^ and the PFC.^27, 28^ It modulates the neurochemical state of the serotonergic system^30^ and is influenced by regional receptor density and synaptic plasticity.^31^ In humans, carriers of the *TPH2* T allele have been associated with increased risks for psychiatric diseases associated with impaired impulse control,^32, 33^ and disturbed affective behavior.^27, 34, 35, 36^ With regard to the serotonergic modulation, we based our hypotheses on findings by Worbe *et al.*^9^ expecting to find an interaction between *TPH2* genotype and impulsivity, for example, in terms of a strong serotonergic modulation in highly impulsive T allele carriers.

## Materials and methods

### Subjects

We examined 103 male students aged from 19 to 28 years (24.0±2.6 years). Subjects were recruited at the University of Wuerzburg, Germany, and were all of Western European descent. The sample size exceeded the minimal sample size of *n*=60 for repeated measures analysis of variance (ANOVA) models with within–between interaction as determined by G*Power (http://www.gpower.hhu.de/). All subjects were screened for impulsivity using the ‘impulsivity scale' of the Wender-Reimherr-Interview and the scale of ‘hyperactivity and impulse control' of attention-deficit/hyperactivity disorder checklist.^37^ Right-handedness was ascertained using the Edinburgh Handedness Inventory.^38^ The study was approved by the ethics committee of the Faculty of Medicine, University of Wuerzburg, and was conducted in accordance with the Declaration of Helsinki in its latest version from 2008. Written informed consent was obtained from all subjects.

### Genotyping

Genomic DNA was extracted from whole-blood samples according to a standard desalting protocol. Genotyping procedures were performed using PCR and gel electrophoresis. Genotyping for the functional tryptophan hydroxylase-2 (*TPH2*) G/T) variant (rs4570625) was performed according to the published protocols.^34, 39^ Genotypes were determined by investigators blinded for phenotypes and independently by two investigators. *TPH2* genotype distribution (TT=3, 4.3% GT=36, 33.4% GG=64, 65.3% *P*(Exact)=0.56) did not significantly differ from the expected numbers calculated according to the Hardy–Weinberg equilibrium using the program DeFinetti provided as an online source (http://ihg.gsf.de/cgi-bin/hw/hwa1.pl).

Based on the findings showing that TPH2 expression is decreased in carriers of the G allele^40^ and in accordance with several previous studies investigating its functional impact,^27^ we defined two groups as follows: (a) subjects homozygous for the *TPH2* G allele (*n*=64) and (b) carriers of at least one T allele (*n*=39). In accordance to these findings, we assumed a progressive allele model in comparing *TPH2* T allele carriers with GG homozygotes in all statistical analyses.

### Experimental paradigm

The used paradigm was an adapted version of the four-choice serial reaction time task by Voon *et al.*^8^ The task consisted of one baseline run outside the scanner and five experimental runs within the scanner.

**Figure 1:**
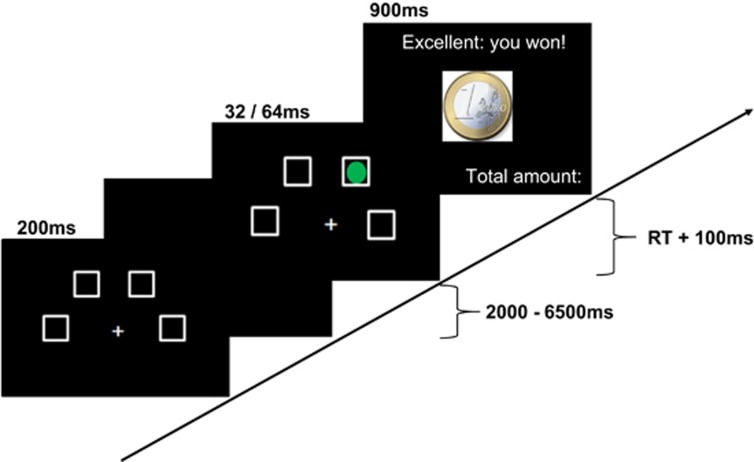
Represents one exemplary experimental trial.

In the task, subjects were instructed to detect a brief visual target after a waiting period to earn a monetary reward. An experimental trial included the following phases/experimental conditions starting with the ‘cue' presentation, with the cue representing the start signal and initiating the waiting period (cue-target interval). In contrast to the behavioral task, where subjects had the space bar to keep pushed along the waiting interval, the start signal in the functional magnetic resonance imaging version was only a visual cue without a following motoric action, due to the minimization of motor artifacts. The second condition was the ‘target' onset, the presentation of a green circle in one of the choices and was followed by the subjects response. The trial ended with the reward feedback (‘reward' condition): according to the subject's performance, a reward/punishment was administered (Figure 1), showing the amount of recently earned/lost money in combination with the overall amount of earned money. The subjects were instructed to press the corresponding button as fast and as correct as possible (Figure 1).

A scanning session included the following steps: outside the scanner, all subjects underwent two training sessions of 10 trials each and a baseline run of 20 trials. To do so, the subjects were seated in front of a computer monitor with a keyboard in front of them (in contrast to touch pad version). In the scanner, subjects lay with response devices in their lap, (Response Grip by Nordic Neuro Lab http://www.nordicneurolab.com/). The baseline run outside the scanner had a duration of 2.5 min, the part within the scanner a total duration of 14 min.

Over the course of five runs, WI was manipulated by the following:

(a) Implementing a monetary reward: (i) a 1 Euro gain when subjects answered extraordinarily fast and correct, (ii) a 10 Cent win when the subjects reacted in their average velocity and correct and (iii) the loss of 1 Euro when subjects reacted too slow. Incorrect responses did not have consequences. The criteria for the decision of extraordinarily fast/average/too slow was determined individually in the first baseline run outside the scanner. In this baseline runs, no reward was implemented and it served the determination of the individual RT in correct responses. The mean RT_M_±s.d. was defined as follows: the RT= RT_M_±s.d.→+10 Cent, RT<RT_M_±s.d.→+1 Euro and RT=>RT_M_±s.d.→−1 Euro.

(b) Manipulating the target's presentation duration from 64 ms in the first three experimental runs to 32 ms in the latter runs.

(c) Varying of the cue-target interval: whereas in the first two runs the cue-target interval was fix (2000 ms), the duration varied in the last three runs between 2000 and 6500 ms.

(d) Including distractor targets in the last experimental runs in terms of targets with blue and/or yellow circles preceding the actual target.

### MR—data acquisition

Scanning was performed on a 3 Tesla TIM Trio Scanner (Siemens, Erlangen, Germany). Whole-brain T2*-weighted BOLD images were recorded with a gradient echo-planar imaging sequence (repetition time=2000 ms, echo time=30 ms, 36 slices, 3 mm thickness, field of view=192 mm, flip angle=90°, 425 volumes). In addition, an isotropic high-resolution T1-weighted three-dimensional structural magnetic resonance (MR) image was acquired (magnetization prepared rapid gradient echo, 176 slices, 1 × 1 × 1 mm^3^, repetition time=2400 ms, echo time=2.26 ms, field of view=256 mm, flip angle=9°).

### MR—data processing

Data processing was performed using the Statistical Parametric Mapping Software Package (SPM12, Wellcome Department of Imaging Neuroscience, London, UK, Wellcome Trust Centre for Neuroimaging; http://www.fil.ion.ucl.ac.uk/spm/). Data preprocessing in the native space included the steps of temporal and spatial alignment: all images were slice time corrected, realigned to the first functional image and unwarped. Images were then spatially normalized into a standard stereotactic space (Montreal Neurological Institute), resampled to an isotropic voxelsize of 2 × 2 × 2 mm^3^ and spatially smoothed with a Gaussian kernel of 8 mm full width at half maximum.

Statistical analysis on the individual first level (single subject level) was based on the general linear model (GLM) approach. Model specification included the definition of experimental condition, in our case ‘cue', ‘target' and ‘reward', whereas reward trials were subdivided into ‘reward:win' and ‘reward:loss' trials. Break periods were defined as ‘rest'. In addition to the experimental conditions, nuisance regressors were specified, that is, ‘error trials' and ‘realignment parameters' (that is, six regressors containing movement in three spatial and three rotational axes), to correct for error variance and movement artifacts. For each condition, onset times were determined from log-files with onsets of the cue condition were determined at the time when the cue picture was presented. Onset times of target trials were defined in terms of the appearance of the target picture and onset times of reward trials (win and loss) were the time points when the reward feedback picture appeared on the screen. The onsets of error trials were defined as the target onsets of incorrect trials. On the single subjects, three contrasts of interest were calculated, ‘cue>rest' to identify cue-specific brain activation, ‘target>rest' to isolate target-induced brain activation and ‘reward' in terms of ‘win>loss' to identify brain activation associated with the receipt of monetary reward. Resulting contrast images entered statistical group analysis.

### Statistical analysis—GLM

On the group level, a repeated measure ANOVA was defined using the within-subject factor conditions (cue vs target vs reward) as independent factor and contrast images as dependent variables. Statistical analyses were performed for the whole brain and in a region of interest (ROI)-based approach focusing on brain activation in the vmPFC and the NAcc. Mask images were used from the WFU Pick atlas (Version 3.0.5b) toolbox,^41^ IBASPM 71 atlas:^42^ nucleus accumbens left/right and medial fronto-orbital gyrus left/right for the vmPFC. Results were reported using family-wise error correction with *P*<0.05.

### Statistical analysis—DCM

For DCM analysis, we used DCM 12 as implemented in the SPM12 software. In the present project, DCM analysis focused on the interplay of the vmPFC and the NAcc addressing its endogenous connections and the condition-specific modulation of the regions and their connections (modulatory inputs). The choice of subject-specific coordinates will be guided by ROI-based group activation maxima in the two network regions from GLM results (see the Results section) with the exact coordinates being determined by averaging coordinates across condition. Volume of interest spheres with a radius of 5 mm were built around the averaged coordinates in the NAcc (*x*=12, *y*=9, *z*=−12) and with a radius of 8 mm in vmPFC (*x*=7, *y*=55, *z*=−11). Different sphere sizes were chosen due to the regional volume size of the structures. Regional time series were extracted as the first eigenvariate of all network regions for the conditions ‘cue', ‘target' and ‘reward', and adjusted for the effects of interest.

**Figure 2:**
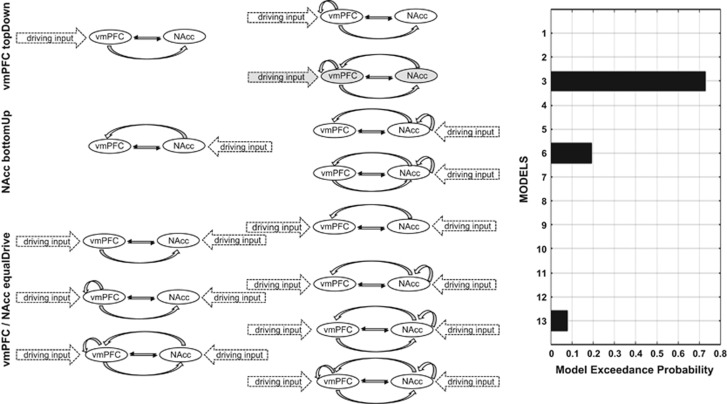
Dynamic casual models that entered BMS model comparisons. On the left, the names of the model families are presented; in the center, all models are shown with black solid arrows indicating endogenous connectivity, gray curved arrows representing condition-specific modulatory niput and arrows with dotted lines illustrate the driving input. The barplot on the right displays the model exceedance probability, resulting from Bayesian Model comparison. BMS, Bayesian model selection; NAcc, nucleus accumbens; vmPFC, ventromedial prefrontal cortex.

Based on introduced findings, three model families were constructed. In family one (NAcc bottom-up), it was assumed that the NAcc drives connectivity between the NAcc and vmPFC condition specifically. In this family, it is assumed that the interplay between the vmPFC and NAcc during WI is predominantly influenced by reward- and satisfaction-driven NAcc activity. In family two (vmPFC top-down), the modulatory connection from the vmPFC to NAcc was assumed being predominantly driven by the vmPFC in terms of frontal top-down modulation. Models of this family imply a well-controlled WI performance based on a strong impulse control by the vmPFC. In family three (vmPFC\NAcc equalDrive), both structure drive network connectivity comparatively (for all families and model, see Figure 2). Models of this family assume a balanced interplay between the influences of the vmPFC and NAcc while performing the 5-CSRTT. Model connections were systematically varied between networks regions.

The families covering 13 models were compared applying random-effects Bayesian model selection^43, 44^ within a pre-specified Occam's window (*P*<0.05). Individual parameter estimates of the model with highest evidence were then assessed by means of random-effects Bayesian model averaging^45^ across the models of the winning family. The Bayesian model averaging parameter estimates were then entered into summary statistics at the group level. The significance of each parameter was assessed by a one-sample *t*-test at a statistical threshold of *P*<0.05, FDR- corrected to account for multiple comparisons.^46^ To address condition-specific modulation of connectivity, repeated measure ANOVA models were defined with the within-subject factor conditions (endogenous connectivity vs cue-specific modulation, vs target-specific modulation vs reward-specific modulation), for each connection respectively (NAcc→vmPFC, vmPFC→Nacc). *Post hoc* paired *t*-tests were, finally, performed to identify significant modulation. Threshold for statistical significance was, as mentioned above, *P*<0.05, FDR-corrected for multiple comparisons.

### *TPH2* genotype-by-impulsivity interactions

To address the influence of both *TPH2* genotype and impulsivity on connectivity between the NAcc and vmPFC, 2 × 2 ANOVA models were defined. As mentioned before, *TPH2* genotype groups were defined as T allele carriers and GG homozygotes. The between-subject factor impulsivity classified subjects with a number of premature responses ⩾3 in the 5-CSRTT as high impulsive subjects and subjects with number of premature responses <3 as low impulsive subjects. The threshold of 3 was chosen as it represented the median value of the range of premature responses across all subjects (range: 0–6 number of premature responses, adapted from Feja *et al.*^19^).

To reveal the impact of *TPH2* genotype and impulsivity on condition-specific modulation, 2 × 2 × 4 repeated measure ANOVA models were performed using the independent factors *TPH2* genotype and impulsivity, and the within-subject factor condition-specific modulation (endogenous connectivity vs cue-specific modulation, vs target-specific modulation vs reward-specific modulation). Threshold for statistical significance was *P*<0.05, FDR-corrected for multiple comparisons.

## Results

Experimental groups did not differ significantly with regard to age and clinical questionnaires (for details, see Table 1). By definition, high impulsive subjects committed significantly more premature responses than low impulsive subjects.

### Behavioral data

The detection for outliers revealed one subject with high number of errors/low accuracy and one subject with low gain of reward. Normal distribution was examined using the Kolmogorov–Smirnoff test, confirming a normal distribution for reaction times (rt_bl1: *P*=0.06; rt_bl2: *P*=0.2; rt_reward: *P*=0.09), reward (win_0.1: *P*=0.2; win_1.0: *P*=0.09; total win: *P*=0.2) and motivation index (*P*=0.18). With regard to accuracy measures, only accuracy (%_correct) was normally distributed (lateRes_%: *P*=0.2).

Significant genotype-by-impulsivity interaction in baseline RT was found with high impulsive T allele carriers being significantly slower than high impulsive GG homozygotes (high impulsive T allele carriers: 395±7, high impulsive GG homozygotes: 371±7, *t*=3.1, *P*<0.05). With regard to all other behavioral parameters, we did not find any significant difference. There was no significant correlation between number of premature responses and any other behavioral parameter. Non-parametric analyses using Mann–Whitney *U*-tests on not normally distributed behavioral parameters did not reveal any significant differences neither between genotype nor impulsivity groups (correct responses (no): *M*_GG_low_=73.4±0.7, *M*_GG_high_=71.6±0.9, *M*_T+_low_=72.1±0.9, *M*_T+_high_=72.9±1.1, *P*=0.58; incorrect responses (no): *M*_GG_low_=6.3±0.6, *M*_GG_high_=8.6±0.9, *M*_T+_low_=7.3±0.9, *M*_T+_high_=7.1±1.0, *P*=0.77; late responses (no): *M*_GG_low_=19.3±1.5, *M*_GG_high_=18.0±2.2, *M*_T+_low_=17.6±2.2, *M*_T+_high_=15.5±2.4, *P*=0.27; late responses (%): *M*_GG_low_=19.9±1.3, *M*_GG_high_=19.3±1.9, *M*_T+_low_=18.9±1.9, *M*_T+_high_=16.8±2.1, *P*=0.39).

### Functional magnetic resonance imaging data

In the cue condition, we found frontal activation bilaterally within the medial posterior gyrus (*Z*_left_=14.5, *Z*_right_=12.1), in animals associated with the prelimbic cortex, and the left insula (*Z*=7.5). In addition, subcortical regions such as the pallidum (*Z*_left_=10.7, *Z*_right_=10.7) and the thalamus (*Z*_left_=8.9, *Z*_right_=9.5) were significantly activated and the postcentral gyrus bilaterally within the parietal lobe (*Z*_left_=7.9, *Z*_right_=5.8).

In the target condition, significantly activated regions were located in the medial and lateral frontal lobe (right posterior medial gyrus: *Z*=22.2; right insula: *Z*=24.9; right inferior frontal gyrus: *Z*=25.0), the parietal cortex (superior parietal gyrus: *Z*_left_=26.9, *Z*_right_=22.5) and the thalamus (*Z*=23.9).

**Figure 3:**
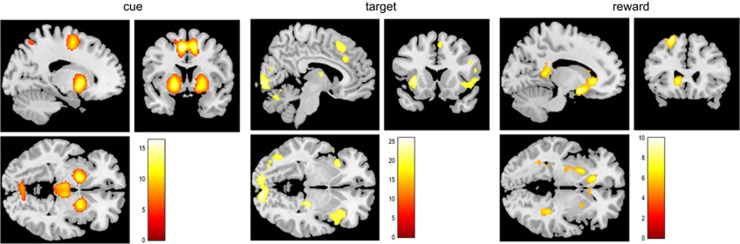
Significant activation patterns for all conditions, cue, target and reward. Statistical threshold for activation patterns was *P*<0.05, corrected for multiple comparisons using family-wise error correction.

The reward condition was associated with increased activation within the left middle frontal gyrus (*Z*=8.8), left and right (para)hippocampal regions (*Z*_left_=7.4, *Z*_right_=7.4) and putamen (*Z*_left_ =7.7, *Z*_right_=5.9). In addition, the NAcc was bilaterally activated (*Z*_left_ =7.7, *Z*_right_=7.2), and the left middle orbital gyrus (vmPFC, *Z*=5.8; for all GLM results, see Table 2 and Figure 3).

**Figure 4:**
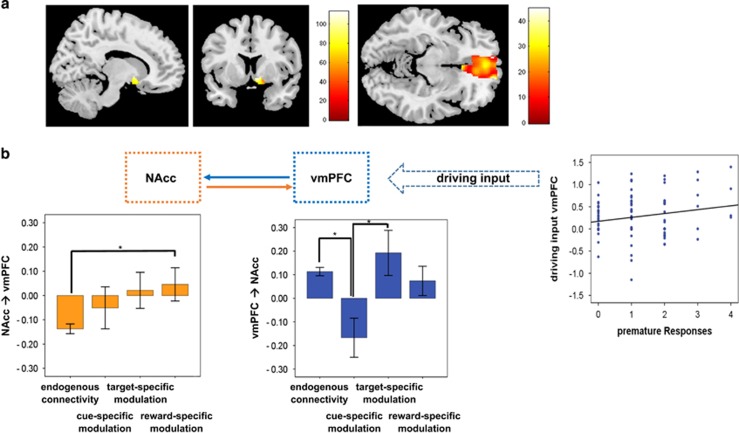
(**a**) In the upper row, brain activation in the NAcc and the vmPFC superimposed on a single subject anatomical image. Color bars represent F-scores as revealed by a repeated measures ANOVA, *P*<0.05, family-wise error correction for multiple comparisons. (**b**) In the center, the dynamic causal model is represented with squares indication the network regions and the solid arrows the connectivity emerging from one region and going to the second. The dotted arrow represents the driving imput by the vmPFC. Barplots at the right and left end of the lower row represent significant change in connectivity across experimental conditions. Blue represents frontal top-down regions and connectivity and orange reward-related regions and connectivity. The scatterplot shows the significant correlation between the number or premature responses and the driving input by the vmPFC. Statistical threshold for connectivity analyses was *P*<0.05, corrected for multiple comparisons using the false discovery rate as suggested by Benjamini and Hochberg.^46^ ANOVA, analysis of variance; NAcc, nucleus accumbens; vmPFC, ventromedial prefrontal cortex.

Using the ROI analysis, we found that both the NAcc and vmPFC were involved in every condition as follows: (a) cue: *Z*_NAcc_=7.8, *k*=45; no significant vmPFC activation; (b) target condition: *Z*_NAcc_=8.0, *k*=77; *Z*_vmPFC_=10.8, *k*=416; (c) reward condition: *Z*_NAcc_=7.0, *k*=65; no significant vmPFC activation (Figure 4).

### DCM estimates

Model comparison for the whole group favored the vmPFC top-down family with a exceedance probability of × *P*=0.9992 vs × *P*=0.0008. In the winning family, the model with the highest model exceedance probability (× *P*=0.88) included bidirectional endogenous connectivity between both structures, bidirectional modulatory input connectivity between both network regions and intrinsic as well as driving input by the vmPFC. All group-specific model comparisons also favored model 3, except for the high impulsive T allele carriers, who favored a model with a driving input by both structures, the vmPFC and NAcc (Supplementary Table S1).

The one-sample *t*-test, addressing connections of significant endogenous connectivity strength revealed that the NAcc and vmPFC were significantly connected in both directions (NAcc→vmPFC: −0.14±0.02, *T*=6.8, *P*<0.01; vmPFC→NAcc: 0.11±0.02, *T*=6.4, *P*<0.01). In addition, a significant driving input was found for the vmPFC (27±0.04, *T*=6.0, *P*<0.01). With regard to the signature, we found that connectivity associated with the vmPFC (that is, driving input and endogenous connectivity) was negative, which hinted towards an inhibitory or controlling influence, endogenous connectivity coming from the NAcc and going to the vmPFC was positive/excitatory. Finally, connectivity behavior correlations revealed that the driving input of the vmPFC was significantly correlated with the number of premature responses (*r*=0.198, *P*<0.05).

**Figure 5:**
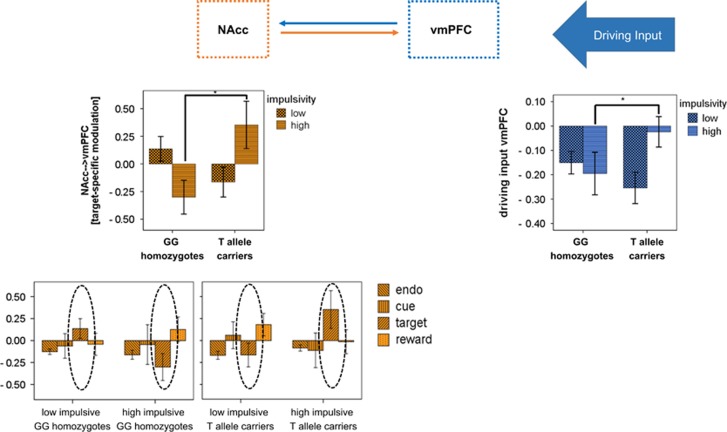
Significant results from *TPH2* genotype-by-impulsivity interactions. In the upper row, the dynamic causal model is represented bar plots at the right and left end of the lower row represent significant *TPH2* genotype-by-impulsivity interactions in connectivity across experimental conditions. Blue frontal top-down regions and connectivity, and orange represents reward-related regions and connectivity. Statistical threshold for connectivity analyses was *P*<0.05, corrected for multiple comparisons using the false discovery rate as suggested by Benjamini and Hochberg.^46^ NAcc, nucleus accumbens; vmPFC, ventromedial prefrontal cortex.

In the condition-specific DCM analysis using a repeated measure ANOVA with the within-subject factor of condition (endogenous connectivity vs cue-specific modulation, vs target-specific modulation vs reward-specific modulation), we found in the modulatory input starting from the NAcc and going to vmPFC a steady increase in connectivity across the conditions with a significant increase in the excitatory influence of the NAcc on the vmPFC during the reward condition. The vmPFC in return showed a significant change in modulation during the cue condition in terms of a significant inhibition of the NAcc followed by a significant excitatory modulation of the NAcc during the target condition (for details, see Table 3 and Figure 5).

In a 2 × 2 ANOVA model with the factors *TPH2* genotype Table 4 (GG homozygotes vs T allele carriers) and impulsivity (high vs low impulsive subjects), we did not find any significant difference neither between *TPH2* genotypes (GG homozygotes vs T allele carriers) and nor between low and high impulsive subjects. However, involvement of the vmPFC was found to be altered in the high impulsive T allele carriers (*TPH2* genotype-by-impulsivity interaction): in T allele carriers, driving input of the vmPFC was significantly reduced in high impulsive T allele carriers compared to low impulsive T allele carriers hinting towards a reduced top-down control in high T allele carriers.

In a 2 × 2 × 4 repeated measure ANOVA addressing genotype-by-impulsivity by condition-specific modulation interactions, we found a significant condition by *TPH2* genotype-by-impulsivity interaction the way that target-specific modulation emerging from the NAcc and heading towards the vmPFC (NAcc→vmPFC) was significantly enhanced in high impulsive T allele carriers: whereas in the low impulsive subjects, no *TPH2* effect was significant, target-specific modulation of the vmPFC by the NAcc was significantly higher in the high impulsive T allele carriers compared with the high impulsive GG homozygotes. In addition, in high impulsive GG homozygotes, modulation was rather inhibitory; T allele carriers, however, showed an excitatory modulation of the vmPFC by the NAcc hinting towards an enhanced anticipation of reward in the high impulsive T allele carriers in the target condition.

## Discussion

In this study, we examined the serotonergic modulation of WI in humans. We applied the human version of the 5-CSRTT in the MR scanner and found WI-associated brain activation patterns in line with findings from animal^6^ and human studies.^13, 47, 48, 49^ Performing effective connectivity, we focused on the interplay between the vmPFC and NAcc, and found inhibition-related and reward-specific alterations in the vmPFC and NAcc. Finally, we investigated the serotonergic modulation on effective connectivity by comparing *TPH2* rs 4570625 GG homozygotes with T allele carriers and a *TPH2* genotype × impulsivity interaction with high impulsive individuals being defined as individuals with a high number of premature responses compared with low impulsive individuals (individuals with few premature responses).

### WI in humans—neural activation patterns

To date, WI as measured via the 5-CSRTT has predominantly been examined in animals. A very detailed model of neural structures associated with WI, thus, relies on animal findings and involves, as introduced, frontal regions covering the vmPFC, ACC, ventral and dorsal prelimbic and infralimbic cortices, mediotemporal regions, and the subcortical structures NAcc. In a strikingly similar way, human subjects in our study activated the same network, although regional activation varied across experimental conditions. For example, highest PFCrecruitment of (human-specific) dorsolateral and ventromedial localization was found during ‘target' and ‘reward' processing.

Target processing has been associated with a high demand of controlling and inhibition, as the restrain of action accumulated over the course of the waiting period.^4, 7^ Top-down control in humans has been crucially associated with the dorsolateral PFC^50^ solely but also in combination with parietal regions, as it was also the case in our study. Fronto-parietal activation preserves the initiation and the adjustment top-down control.^51^

In the reward context, in return, fronto-parietal pathways have been linked to temporal delay of gratification^52^ in terms of a linear relation between fronto-parietal recruitment and degree of delay discounting.^53^ PFC activation during reward processing in the vmPFC has been implicated in reward representation and reward prediction,^49, 54, 55^ with reward representation involving processes of coding the stimulus reward value and guidance of action selection for reward.^55^ Similar observations were made in animals and the infralimbic cortex.^56, 57, 58^ The additional dorsolateral PFC recruitment, however, seems to be rather specific to human and has been discussed in the context of reward feedback evaluation^59, 60^ and self-regulatory processes in response to rewarding stimuli.^61^ Finally, frontal activation subsumed also cingulate regions (prelimbic cortex) and predominantly in the impulsivity-associated conditions ‘cue' and ‘target'. Prelimbic cortices have strongly been related to inhibition, for example, in a spatial conditioning task inactivation of prelimbic regions did lead to increased responding in rats^62^ without affecting learning and consolidation. In humans, the cingulate cortex together with PFC has been described as regulators of conflict detection and behavioral inhibition, in paradigms with and without aspects of delay discounting.^5^

The second crucial structure in the model of WI is the NAcc, demonstrating strongest involvement in the reward condition. As introduced the NAcc is the key structure of the mesolimbic reward system^63, 64, 65^ in both humans and animals, and has been shown to specifically modulate behavior in the 5-CSRTT,^66, 67^ modulating behavior in the expectation of the reward. Similar cognitive mechanisms have been found also in humans as ROI-based analyses revealed significant activation across all conditions, ‘cue', 'target' and ‘reward'.

Finally, the model highlights mediotemporal structures such as the amygdala and hippocampus.^68^ Functionally, the hippocampus has been discussed as reflecting reward prediction and prospective evaluation of future outcomes. Lesion studies showed that hippocampal damage in rats led to an increase in delay discounting capacities, however, in combination with an increase in impulsive behavior.^69, 70^ In our human sample, we found an increase in hippocampal activation in the reward condition, most probably reflecting prediction and outcome processing.

In contrast to the model, we did not find significant activation in the ACC in human young adults. Functionally, the ACC has been related to error monitoring and conflict processing.^71^ As the task was very easy for young adults, the lack of ACC recruitment might therefore be based on the lack of the demand to this cognition. Therefore, we conclude that the animal-based neural model fits astonishingly well to human activation findings, hinting towards similar cognitive processes across species.

### The interplay between the NAcc and vmPFC in humans—condition-specific variation and its modulation by TPH2 and impulsivity

In addition to whole-brain analyses, we focused on the interplay between the NAcc and vmPFC in 5-CSRTT processing. For an accurate quantification of this interplay, we chose effective connectivity using the DCM approach.^25^

Model comparison showed that for the whole group, a model including bilateral connections between the NAcc and vmPFC best fitted the data, which was predominantly driven by the vmPFC. We found that modulatory input of the NAcc increased over the course of one trial with a strongest excitatory modulation during the reward receipt. In contrast, inhibitory modulation by the vmPFC was strongest before target presentation that changed into an excitatory modulation at target presentation. Finally, we found a significant correlation between the vmPFC driving input, and the number of premature responses proving the role of the vmPFC in the control of impulses. In line with the findings by Donelly *et al.*, we found that connectivity emerging from the NAcc was highest during the reward condition, indicating that the impact of the NAcc on the vmPFC was strongest during reward processing (in comparison with all other conditions).

In addition to similarities in NAcc response in rats and humans, we found that the vmPFC showed increased connectivity during target condition. However, the impact of the vmPFC on the NAcc in humans seemed to be more complex: whereas the vmPFC-based connectivity was strongly negative during the cue condition at the beginning of the experimental trials describing an inhibiting influence of the NAcc by the vmPFC, connectivity significantly increased during target condition, thus having an impact on excitatorily the NAcc. On the cognitive level, inhibitory influence at the beginning of the trial might confer earlier described outcome-oriented processing in humans with the vmPFC subserving the top-down control of the NAcc during an early stage of the trial processing. The need inhibitory control ended with correct target processing, reversing the inhibitory control into an excitatory influence of the NAcc ‘allowing' the anticipation of reward.

Genetic analyses showed that serotonergic modulation of NAcc–vmPFC modulation was dependent on the individuals impulsivity. Applying *TPH2* genotype-by-impulsivity interactions, we found that vmPFC top-down control was reduced in high impulsive *TPH2* T allele carriers, as revealed in combination with increased reward anticipation behavior during target processing. Serotonergic modulation has proven to have an important role in action withholding such as WI and deferring gratification,^72, 73^ probably affecting the motivational significance of the pre-potent action to be inhibited on the basis of future reward or punishment,^74, 75^ as shown in animal^76, 77^ and human studies.^78, 79, 80^ *THP2* has furthermore been shown to influence impulsive behavior; genetic association between the *TPH2* gene and/or TD and impulsivity and with the impulsivity-associated neuropsychiatric disorder attention-deficit/hyperactivity disorder has repeatedly been reported.^81, 82, 83, 84, 85, 86, 87^ For example, Stoltenberg *et al.*^85^ examined 199 college students performing a computerized stop signal task. They found that performance varied in terms of individuals with the T/T genotype showing the longest RTs. The authors concluded that individuals with the T/T genotype may have a reduced *TPH2* function and correspondingly lower central serotonin levels resulting in higher impulsivity.^85^ Likewise, Oades *et al.*^82^ found that an under-transmission of the A-allel of SNP rs6582071 was associated with behavioral impulsivity.^82^

On the physiological level, *TPH2* is also very closely linked with the mesolimbic reward system. For example, Carkaci-Salli *et al.*^88^ showed high TPH2 activity and protein expression (second highest after the raphe nuclei) was present in the ventral tegmental area including the NAcc.^88^ Pharmacological manipulation of central serotonin showed the dose-dependent effects on reward processing: whereas a single low dose of the selective serotonin reuptake inhibitor (SSRI) citalopram increased reward sensitivity, a single high dose had the opposite effects.^89^ Thus, the enhanced reaction to reward in combination with impaired cognitive control in T allele carriers is in line with earlier findings.

## Limitations and conclusion

To our knowledge, this is the first study examining the neural underpinnings of WI in humans addressing its serotonergic modulation. The concept of WI, to date, is mainly a theoretical construct and has barely been used in empirical impulsivity studies in humans. In addition, neural findings recorded while the 5-CSRTT are sparse and restricted to the vmPFC and NAcc. Thus, findings of both the involved cognitive processes and associated brain regions are not well known. Therefore, GLM brain activation analyses in this study had to be performed in an exploratory than hypothesis-driven approach. In addition, connectivity analyses were restricted to only two regions, whereas there are many more brain regions involved in the processing, as shown by the GLM analyses on whole-brain level. However, we chose this paradigm as well as the network regions for DCM analyses for our pilot study to examine its potential for translational studies with regard to its aptness with regard to cognitive and neural functions. Based on the high overlap between the current findings with animal reports from the level of cognitive processes, over activation of the brain network of WI as described by Dalley *et al.* ^6^ up to the interplay between the two (anatomically small) key regions NAcc and vmPFC by Donelly *et al.*, we conclude that WI as measured by the 5-CSRTT is a promising paradigm for translational studies.

Finally, in contrast to earlier studies, we did not find any significant differences between genotype groups independent of the impulsivity; neither on the behavioral level nor with regard to their impulsivity as measured by the clinical questionnaires or in the neural data in terms of effective connectivity parameters. This might be based on our homogenous sample of male students, aged from 19 to 28 years and ~95% of German origin and education. Therefore, further investigation with a larger sample as well as with effective connectivity analyses on larger networks might be of high scientific interest.

## Figures and Tables

**Table 1:** Description of experimental groups and behavioral data

	*TPH2 GG homozygotes*		*TPH2 T allele carriers*		*Statistics*		
	*Low impulsive (*n*=43)*	*High impulsive (*n*=21)*	*Low impulsive (*n*=21)*	*High impulsive (*n*=17)*	*F*_*genotyp*_	*F*_*impulsivity*_	*F*_*geneXimp*_
Age (years)	24.0 ±2.0	22.9 ±1.9	24.2 ±2.0	23.8 ±1.6	2.0	3.3	0.8
							
*Main behavioral parameters*							
Premature responses (no)	0.2 ±0.5	2.5 ±0.7	0.5 ±0.5	2.8 ±1.7	0.7	124.8**	1.1
Accuracy (% correct)	92.1±0.8	89.5±1.1	90.8±1.1	91.1±1.2	0.1	0.6	1.7
Motivation index (bl2–bl1)	0.03±.01	0.04±.01	0.03±.01	0.05±.01	1.0	2.7	0.5
							
*Descriptives of impulsivity*							
WRI	1.3±1.5	1.7±1.8	1.2±1.4	1.6±1.6	0.06	1.1	0.01
ADHD-CL	5.7±3.9	7.2±4.7	5.8±4.4	6.4±5.9	0.1	1.2	0.2
							
*Behavioral data*							
RT_bl1 (ms)	381±5	371±7	376±7	395±7	2.7	0.3	5.2*
RT_bl2 (ms)	360±5	347±8	355±8	358±9	0.2	0.9	1.3
win_0.1 (no)	26.6±1.2	24.1±1.7	24.5±1.7	23.2±1.9	0.7	2.7	0.1
win_1.0 (no)	27.6±1.8	31.2±2.6	30.1±2.6	34.1±2.9	1.1	3.9	0.0
Total win (Euro)	11.2±3.0	16.2±4.3	15.5±4.3	21.5±4.7	1.3	2.5	0.1
RT_reward presentation	381±6	368±9	372±9	380±10	0.5	0.2	1.6

Abbreviations: ADHD, attention-deficit/hyperactivity disorder; ADHD-CL: ADHD checklist; bl1: baseline run 1; bl2: basline run 2; FDR, false discovery rate; gene, factor genotype; imp, factor impulsivity; RT, reaction time; WRI, Wender-Reimherr-Interview; win_0.1: win of 0.1 Euro; win_1.0: win of 1 Euro.

*P<0.05 FDR-corrected for multiple comparisons; **P<0.01 FDR-corrected for multiple comparisons.

Scores are reported as mean±s.e.

**Table 2:** Condition-specific brain activation, revealed by 1 × 3 ANOVA model, *n*=103

*Hemisphere*	*Region*	k	X	Y	Z	T
*Positive effect of cue*						
Frontal lobe (medial)						
Bilateral	Posterior medial gyrus	3545	−8	4	50	15.4
	Middle cingulate cortex/PLd		10	4	52	12.9
Left	Insula	161	−34	16	10	7.8
Subcortical						
Bilateral	Pallidum	808	−18	6	−4	11.3
		744	20	6	−2	11.3
Bilateral	Thalamus	746	6	−16	−2	10.0
			−4	−16	−2	9.5
Parietal lobe						
Bilateral	Postcentral gyrus	255	−38	−30	42	8.3
		50	40	−28	40	5.9
Occipital						
Bilateral	Lingual gyrus	240	−4	−78	0	7.3
			5	−75	2	5.9
Left	Precuneus	43	−14	−64	56	6.0
						
*Positive effect of target*						
Frontal lobe (medial)						
Right	Posterior medial gyrus/PLd	517	4	22	48	23.8
Bilateral	Insula	1628	36	24	−6	26.5
Subcortical						
Bilateral	Thalamus	72	24	−24	−6	25.3
Frontal lobe (lateral)						
Bilateral	Inferior frontal gyrus/dorsolateral PFC	155	40	36	12	22.2
		1552	−46	6	28	26.7
Parieto-occipital						
Bilateral	Fusiform gyrus	17132	28	−78	−12	33.2
Bilateral	Superior parietal gyrus		−28	−48	44	28.3
			30	−54	18	
						
*Positive effect of reward*						
Frontal lobe (medial)						
Bilateral	NAcc	1045	−12	8	−14	8.2
	Putamen		−24	6	8	
	Nacc	309	12	8	−16	7.5
	Putamen		22	10	−12	
Left	Middle orbital gyrus/vmPFC	426	−2	48	−12	7.1
Mediotemporal						
Bilateral	(Para)hippocampal	701	−14	−42	12	7.5
		1016	32	−40	2	6.9
Frontal lobe (lateral)						
Left	Middle frontal gyrus/dorsolateral PFC	337	−22	28	58	9.2

Abbreviations: ANOVA, analysis of variance; NAcc, nucleus accumbens; PFC, prefrontal cortex; PLd, dorsal prelimbic cortex; vmPFC, ventromedial prefrontal cortex.

Coordinates were reported in Montreal Neurological Institute space. Whole-brain analysis, *P*<0.05 family-wise error corrected.

**Table 3:** Results from one-sample *t*-test as well as repeated measures ANOVA with the within-subject factor connectivity type (endogenous connectivity vs cue-specific modulation, vs target-specific modulation vs reward receipt-specific modulation)

*Connection*	*Repeated measures ANOVA*		
	*Condition*	M	*F*
NAcc→vmPFC	endo	−0.14±0.02	6.1*
	mod_cue	−0.05±0.09	
	mod_target	0.02±0.07	
	mod_reward	0.05±0.07	
vmPFC→NAcc	endo	0.11±0.02	5.0*
	mod_cue	−0.17±0.08	
	mod_target	0.19±0.10	
	mod_reward	0.07±0.06	

	Post hoc** t*-tests*	
*Connection*	*Paired variables*	T
NAcc→vmPFC	endo vs mod_cue	1.1
	endo vs mod_target	2.1
	endo vs mod_reward	2.5*
	mod_cue vs mod_target	0.6
	mod_cue vs mod_reward	0.9
	mod_target vs mod_reward	0.2
vmPFC→NAcc	endo vs mod_cue	3.3*
	endo vs mod_target	0.8
	endo vs mod_reward	0.6
	mod_cue vs mod_target	2.9*
	mod_cue vs mod_reward	2.3
	mod_target vs mod_reward	0.3

Abbreviations: ANOVA, analysis of variance; endo, endogenous connectivity; mod, modulation; NAcc, nucleus accumbens; vmPFC, ventromedial prefrontal cortex.

*P<0.05 false discovery rate-corrected for multiple comparisons.

The values are expressed as mean±s.e.

**Table 4:** Results from 2 × 2 ANOVA model using the factors *TPH2* genotype (GG homozygotes vs T allele carriers) and impulsivity (high vs low impulsive subjects) as factors as well as 2 × 2 × 4 repeated measures ANOVA with the factors TPH2 genotype, impulsivity and connectivity type (endogenous connectivity vs cue-specific modulation vs target-specific modulation vs reward-specific modulation)

	*TPH2 GG homozygotes*		*TPH2 T allele carriers*		*Statistics*				
	*Low impulsive*	*High impulsive*	*Low impulsive*	*High impulsive*	*F*_*gene*_	*F*_*imp*_	*F*_*geneXimp*_	*F*_*con*_	*F*_*gene × imp × con*_
endo: NAcc→vmPFC	−0.13±0.03	−0.16±0.05	−0.19±0.05	−0.8±0.05	0.2	0.3	1.9		
endo: vmPFC→NAcc	0.15±0.03	0.10±0.04	0.10±0.04	0.06±0.04	1.7	1.2	0.1		
mod_cue: NAcc→vmPFC	−0.06±0.14	−0.05±0.19	0.06±0.19	−0.11±0.22	0.1	0.2	0.3		
mod_cue: NAcc→vmPFC	−0.04±0.13	−0.36±0.18	−0.29±0.18	−0.03±0.20	0.1	0.1	2.7		
mod_target: NAcc→vmPFC	0.14±0.11	−0.30±0.16	−0.16±0.16	0.35±0.18	1.3	0.1	9.6*		
mod_target: vmPFC→NAcc	0.11±0.15	0.25±0.22	0.25±0.22	0.22±0.24	0.1	0.1	0.2		
mod_reward: NAcc→vmPFC	−0.04±0.11	0.13±0.15	0.18±0.15	−0.01±0.17	0.1	0.1	0.7		
mod_reward: vmPFC→NAcc	0.08±0.10	0.29±0.14	−0.01±0.14	0.01±0.15	1.1	0.6	0.1		
drivingInput: vmPFC	−0.15±0.05	−0.20±0.07	−0.25±0.07	−0.02±0.08	0.3	2.0	4.2*		
									
*NAcc*→*vmPFC*									
endo	−0.13±0.03	−0.16±0.05	−0.17±0.05	−0.08±0.05	0.8	0.1	0.8	1.4	4.2*
mod_cue	−0.06±0.14	−0.05±0.19	0.06±0.19	−0.11±0.22					
mod_target	0.14±0.11	−0.30±0.16	−0.16±0.16	0.35±0.18					
mod_reward	−0.04±0.11	0.13±0.15	0.18±0.15	−0.01±0.17					
									
*vmPFC→NAcc*									
endo	0.15±0.03	0.10±0.04	0.10±0.04	0.06±0.04	0.3	0.2	0.2	1.2	1.1
mod_cue	−0.04±0.13	−0.36±0.18	−0.29±0.18	−0.03±0.20					
mod_target	0.11±0.15	0.25±0.22	0.25±0.22	0.22±0.24					
mod_reward	0.08±0.10	0.29±0.14	−0.10±0.14	0.01±0.15					

Abbreviations: ANOVA, analysis of variance; con, factor connectivity type; endo, endogenous connectivity; gene, factor genotype; imp, factor impulsivity; mod, modulation; NAcc, nucleus accumbens; vmPFC, ventromedial prefrontal cortex.

*P<0.05 false discovery rate-corrected for multiple comparisons.

The values are expressed as mean±s.e.
