# Supplementary material for: Serotonergic modulation of ‘waiting impulsivity' is mediated by the impulsivity phenotype in humans
Source: Transl Psychiatry. 2016 Nov 8;6(11):e940–. doi: 10.1038/tp.2016.210 (PMC5314122; doi:10.1038/tp.2016.210)
Supplement: Supplementary Information [file tp2016210x1.docx]

| **Table s1.** Bayesian Model Selection | | | | |
| --- | --- | --- | --- | --- |
|  | ***TPH2* GG homozygotes** | | ***TPH2* T allele carriers** | |
|  | **low impulsive [n=43]** | **high impulsive [n=21]** | **low impulsive [n=21]** | **high impulsive [n=17]** |
|  | **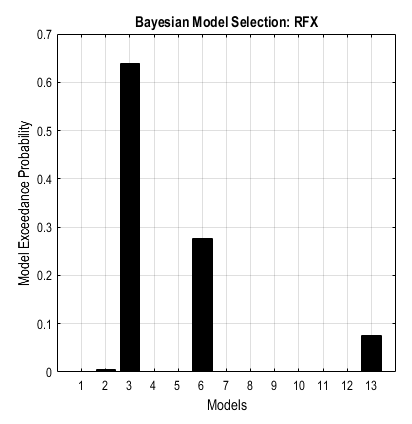** | **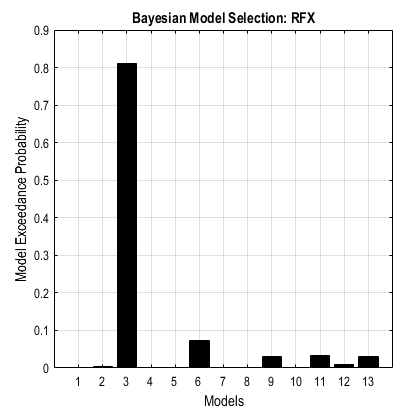** | **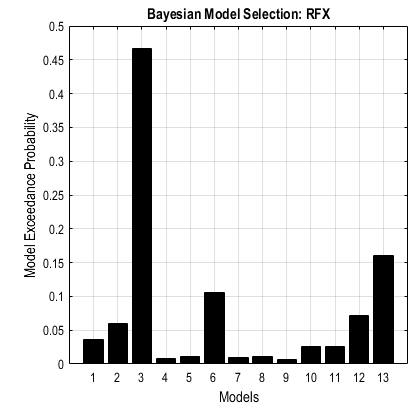** | **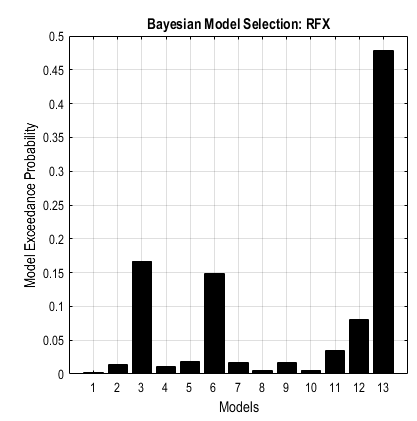** |
| **winning model / xp** | **Model 3 / 0.64** | **Model 3 / 0.81** | **Model 3 / 0.47** | **Model 13 / 0.48** |
|  | | | | |
